# 

**Published:** 1893-02-04

**Authors:** 


**Notices of Births, Deaths, and Marriages**, are inserted in this column at a charge of 2s. 6d. for an announcement not exceeding four lines.

### NOTICE TO CORRESPONDENTS.

All MS., Letters, Books for Review, and other matters intended for the Editor should be addressed THE EDITOR, THE LODGE, PORCHESTER SQUARE, LONDON, W.

The Editor cannot undertake to return rejected M.S., even when accompanied by stamped directed envelope.

**Notice to Advertisers and Subscribers.**

All Advertisements, Orders for Copies of THE HOSPITAL, and Business Communications should be addressed to The Publisher, *not to the Editor*, THE HOSPITAL, 140, Strand, W.C.

The Cost of Subscription, post free, is as follows:—Per week, 2½d.; per quarter, 2s. 6d.; per half-year, 5s.; per annum, 8s. 6d. Foreign:—Per annum, 10s. 8d.; payable, in all cases, in advance.

**"Burdett's Hospital Annual," 1893.**

Fourth year of publication. 700 pp. Boards, gilt lettering. A most complete guide to British, Colonial, Indian, and American Hospitals, Dispensaries, Nursing and Convalescent Institutions, and Asylums. Price 5s. Published by THE SCIENTIFIC PRESS (Limited), 140, Strand, W.C.

**NOTICE.—The Index for Vol. XII. (ending September 1892) is on sale at the Office of this Journal, price 2½d., post free.**

### Scraps and Gleanings.

Mr. Victor Cavendish has been elected President of the Derbyshire Hospital for Sick Children.

Tooting and Streatham Common continue their protests against having fever hospitals in their midst.

Madame Denain, a French actress, has left 250,000 francs towards the establishment of an institution for foundlings.

A friend has kindly promised £500 towards the extension and rebuilding of the Paddington Green Children's Hospital.

The Girdlers' Company have given a donation of £21 in aid of the funds of the Metropolitan Hospital, Kingsland Road, N.

The *Daily Chronicle* hints that the "No Crinoline" agitation will make an especial appeal to H.R.H. the Princess of Wales.

The working men of Hull have started "The Mizpah Gift Fund" to raise £1,000 towards furnishing and equipping the Reckett Convalescent Home.

Mr. William Liddon, who died recently, was one of the most eminent surgeons in the West. He had also obtained a great reputation as an oculist.

The Mayor of Boston has received a cheque for £100 from Lady Ingram Watkin, which sum is to be distributed amongst the poor of Boston and Skirbeck, Lincolnshire.

The Hospitals Medical Association of Paris will award a prize of 1,000 francs to the best work sent in on the "Circulation of the Blood in cases of Infectious Disease."

We hear that H.R.H. the Prince of Wales has expressed his intention of being present at the Royal College of Surgeons on February 14th, when Mr. Thomas Bryant delivers the Hunterian oration.

It is rumoured that Dr. G. Wilkinson, formerly Bishop of Truro, will be nominated to the Scottish Bishopric of St. Andrews, now vacant through the demise of the much-esteemed Dr. Wordsworth.

The Committee of the London Dental Hospital are raising funds for the acquisition of a new site in Leicester Square, the sanitation and accommodation of the old establishment being inadequate.

Herr von Donner is benefiting Hamburg with a Woman's Hospital, in honour of Dr. Michelsen, a woman physician, who treated successfully Frau von Donner through a long and dangerous illness.

The new infirmary at Lancaster is to be proceeded with forthwith. The total cost of the building is estimated at £17,000, exclusive of the munificent gift of Mr. W. Smith, M.P., of £1,000 for a children's ward.

The governors of the Dundee Royal Infirmary acknowledge the receipt of £5[illegible]0, a legacy left by the late Mr. Arklay to the institution also the sum of £100 given by the late Mr. T. H. Cox to the Convalescent Home.

On the anniversary of the death of the Duke of Clarence, the new ward at the Children's Hospital at Dublin was opened by Lord Houghton, who evinces a lively interest in the hospitals of the Irish capital.

At the North-Western Poor Law Conference at Manchester, a resolution in favour of returning to the plank bed at the workhouses, which Mr. Henley had endeavoured to suppress, was carried by 41 votes against 17.

The Hornsey Sanitary Board are considering the necessity of providing the means of isolating cases of infectious diseases. The Board seems disposed to accept the terms and means of isolation which Enfield has offered.

The strongest spring in Lincolnshire has just been discovered at a depth of 245 feet below the surface. The water threw up first tons of blue sand, but now flows over a 2½-in tube 30 feet above the ground at the rate of 4,619 gallons hourly.

A gentleman has promised the sum of £500 to the committee who are raising a memorial to the late Sir William Evans on condition that the subscribers decide to erect a chapel at the New Royal Infirmary at Derby, the cost of which would be £3,000.

The amount of subscriptions already promised towards the new wing to be erected at the Convalescent Home at Cookridge, as a memorial to the late Canon Jackson, is £1,287. The wing is to be for women and children, and is to be called the "Edward Jackson Wing."

As a Christmas gift of thankoffering, Mr. S. M. Burroughs (of the firm of Burroughs and Wellcome, Holborn and Dartford) has promised to give £1,000 towards a cottage hospital for Dartford. Mr. H. M. Stanley has offered to give a lecture in aid of the fund to be raised.

The inhabitants of Great Yarmouth are again agitating against the present site of the Infectious Diseases Hospital. Mr. Harvey-George's suggestion that £300 would be well spent in erecting a new hospital outside the town appears a feasible way of meeting this difficulty.

The Tynemouth Victoria Jubilee Infirmary received during the past year £51 3s. 7d. from the fishing boats of the place. The total income was £852 3s. 3d., and the expenditure amounted to £800 1s. 9d. There were 118 in-patients admitted to the hospital, with an average of 35 days in the house.

The King and Queen of Italy have offered a prize to the inventor of the best apparatus for the conveyance of sick or wounded. The models, with detailed inscriptions, must be sent to Signor L. delli Sanaglia at Rome, where the apparatus will be on exhibition from August till September. A jury of fourteen persons will decide the prize.

Two native gentlemen have provided funds for a New Eye Hospital at Allallabad, and the foundation stone was laid in presence of a picturesque crowd of past patients. Excellent scientific work has been done in the old, most inadequate quarters by Surgeon Lieut.-Colonel Hall, whose reputation as an oculist has attracted sufferers from great distances.

A grand three days' bazaar in aid of the Ulster Hospital for Women and Children was opened in the Ulster Hall by the Duchess of Abercorn. The bazaar represented a Polar scene, with a fleet of exploring vessels "fast bound" in thick, ribbed ice. In addition to "the fleet" there were caves, ice mountains, floes, and crystals; also a large screen depicting one of those brilliant sky illuminations characteristic of the far north.

For Student of Medicine and Medical Appointments see page VI. overleaf.

### THE STUDENT OF MEDICINE.

[All communications for this Supplement should be addressed to The Editor of The Hospital, at 140, Strand, with the words, "Students' Column," written in the left hand top corner of the envelope.]

### VACANCIES.

The following vacancies are announced this week, the amount of salary in each case being given after the name of the institution:

#### Resident Medical Officers.

Albany General Hospital, Grahamstown, South Africa. Resident Medical Officer. Salary £250 per annum, with unfurnished rooms. Further particulars to be obtained of Dr. C. W. Cathcart, Royal Infirmary, Edinburgh.

Boscombe Hospital, Bournemouth. House Surgeon. Salary, £60 per annum, with board and residence. Unmarried. Applications to Honorary Secretary by February 11th.

Cheltenham General Hospital. Junior House-Surgeon, unmarried, doubly qualified. Salary, £40 per annum, board and apartments. Applications to Lieutenant-Colonel Croker King, Honorary Secretary, by February 4th.

Chichester Infirmary, Chichester. House Surgeon. Salary £80 per annum, with board, lodging, and washing. Applications to E. E. Street, Secretary, by March 1st.

Dundee Royal Infirmary. Medical Superintendent. Applications to the Secretary, D. Gordon Stewart, Solicitor, Dundee, by February 8th.

Hulme Dispensary. Dale Street, Stretford Road, Manchester. House Surgeon. Doubly qualified. Salary, £130 per annum, with apartments, coal, and gas. Applications to F. H. Collins, M.D., Secretary Medical Committee, by February 2nd.

Liverpool Infirmary for Children. Assistant House Surgeon. Appointment for six months. Board and lodging provided. Applications to C. W. Carver, Honorary Secretary, by February 6th.

Stockport Infirmary. Junior Assistant House Surgeon. Appointment for six months, with board and residence, and honorarium of £10 at the end of six months' satisfactory service. Applications to the Secretary by February 6th.

#### Non-Resident Medical Officers.

Guy's Hospital, S.E. Assistant Dental Surgeon. Must possess L.D.S. Eng. qualification. Applications to the Dean by February 4th.

University College of South Wales and Monmouthshire, Cardiff. A Professor of Anatomy, and a Professor of Physiology. Stipend in each case £350 per annum. Applications to Ivor James, Registrar, by February 10th.

University of Edinburgh. Additional Examiner in Chemistry. Salary £75 per annum, and £10 allowed for travelling and other expenses if not resident in Edinburgh or neighbourhood. Applications to M. C. Taylor, Secretary, by February 6th.

University of Edinburgh. Additional Examiner in Public Health. Salary £40 per annum, and £10 allowed for travelling and other expenses if not resident in Edinburgh or neighbourhood. Applications to M. C. Taylor, Secretary, by February 6th.

University of Edinburgh. Additional Examiner in Botany. Salary £75 per annum, and £10 allowed for travelling and other expenses if not resident in Edinburgh or neighbourhood. Applications to M. C. Taylor, Secretary, by February 6th.

### PASS LIST.

#### Society of Apothecaries of London.

Primary Examination: Part I. January, 1893.

The following candidates passed in:—

Chemistry.—C. Adams, Royal Free Hospital; W. H. Burnhill; M. K. H. Holst, Royal Free Hospital; H. M. Maitland, Royal Free Hospital; T. H. P. Peers, Charing Cross Hospital; M. Sharp, Royal Free Hospital.

Materia Medica, Botany, and Pharmacy.—W. MacLellan, Edinburgh; J. Thornton, London Hospital.

Part II.

The following candidates passed in:—

Anatomy and Physiology.—F. H. Blatchford, Cambridge and St. Mary's Hospital; H. G. Cribb, Middlesex Hospital; H. C. F. Dalton, Charing Cross Hospital; L. C. Dillon, King's College; A. M. Dow, Leeds Yorkshire College; A. S. Lawrence, Middlesex Hospital; C. R. Maitland, Cambridge University; C. H. R. Pentreath, Cambridge and St. Mary's Hospital; T. E. Price, Birmingham and Mr. Cooke's; W. S. Robinson, Birmingham; C. C. Sibley, Middlesex Hospital.

Anatomy.—W. Alder, Guy's Hospital; L. M. Blake, Royal Free Hospital; D. L. E. Bolton, St. Bartholomew's Hospital; G. H. Gill, Manchester Owens College; S. R. Hallam, St. Thomas's Hospital; H. G. Jones, St. Mary's Hospital; E. S. Johnson, St. Mary's Hospital; E. S. L. Lovell, Charing Cross Hospital; A. C. McLean, King's College; E. D. Warren, University College and Sheffield.

Physiology.—E. H. Betts, St. Bartholomew's Hospital; A. T. Latchmore, Leeds Yorkshire College; W. A. Montgomery, St. Thomas's Hospital; A. P. Square, Middlesex Hospital; M. A. F. Vakil, Royal Free Hospital and Bombay.

